# Phytotherapy Perspectives for Treating Fungal Infections, Migraine, Sebhorreic Dermatitis and Hyperpigmentations with the Plants of the Centaureinae Subtribe (Asteraceae)

**DOI:** 10.3390/molecules25225329

**Published:** 2020-11-15

**Authors:** Joanna Nawrot, Justyna Gornowicz-Porowska, Gerard Nowak

**Affiliations:** Department and Division of Practical Cosmetology and Skin Diseases Prophylaxis, Poznan University of Medical Sciences, 33 Mazowiecka Street, 60-623 Poznań, Poland; joannac@ump.edu.pl (J.N.); justynagornowicz1@poczta.onet.pl (J.G.-P.)

**Keywords:** Asteraceae, sesquiterpene lactones, coumarins, phytoecdysones, arbutin, phytotherapy

## Abstract

Sesquiterpene lactones, coumarins, phytoecdysones and phenolic compounds are characteristic of the species from the subtribe Centaureinae (Asteraceae). Many of the compounds isolated from plants of the Centaureinae subtribe have strong pharmacological properties. It may be suggested that these compounds’ chemical structure might be an indicator of these pharmacological properties. The aim of the study was to describe recent studies in the field of phytotherapy, focusing on compounds isolated from chosen plants of Centaureinae and the possibilities of using them to treat antifungal infections, inhibit serotonin and ease symptoms of seborrhea dermatitis and hyperpigmentation. The results of these biological studies have shown that in the future, extracts from the above-mentioned plant material may be used as active substances in new safe and effective drugs.

## 1. Introduction

For a long time, pharmacologists have been analyzing natural compounds, such as sesquiterpene lactones, coumarins, phytoecdysones and phenol glucosides, isolated from the plants of the Asteraceae family.

Special interest has been given to sesquiterpene lactones because of their strong pharmacological properties [1]. Sesquiterpene lactones isolated from *Matricariae flos*, *Arnicae flos* and *Millefoli herba* have anti-inflammatory effects [2]. It has been established that sesquiterpene lactones can inhibit DTH (Delayed-Type Hypersensitivity test), especially contact dermatitis induced by intratracheal administration of *hapten* [3].

Coumarin compounds isolated from Centaureinae plants may be treated as chemotaxonomy markers for the *Psephellus* genus [4]. 7-hydroxycoumarin (umbelliferone) can absorb UV light in the range of 280–315 nm, and it is therefore used for anti-UV cosmetics production [5], and it is one of the active substances appearing in the root and herb of *Hieracium pilosella* L., a plant with proven antifungal properties [6].

Phytoecdysones are yet another group of compounds appearing in the species of the mentioned subtribe [7], and plants from the *Serratula* genus are their most efficient source [8]. Reliable qualitative and quantitative analyses of phytoecdysteroids isolated from plants are important for the development of new pharmaceutical products [9]. Natural steroids are nowadays often chosen for skin change treatment, replacing synthetic steroids [10].

Phenol glucoside arbutin usually appears in plant extracts along with methyl arbutin and hydroquinone. The first of these two compounds weakens arbutin effectiveness, while hydroquinone’s safety is sometimes questioned [11].

Phytochemistry of plants from the Asteraceae family has been analyzed in the Department of Medicinal and Cosmetic Natural Products of Poznan University of Medical Sciences (now Department and Division of Practical Cosmetology and Skin Diseases Prophylaxis) for over forty years. We have narrowed down our research to the Centaureinae subtribe and managed to analyse such species as *Zoegea baldschuanica* C. Winkl., *Z. leptaurea* L, *Centaurea* sp., *Chartolepis* sp., *Rhaponticum* sp., *Leuzea* sp., *Psephellus bellus* (Trautv.) Wagenitz, *P. sibirica* (L.) Wagenitz, *Stizolophus balsamita* (Lam.) K. Koch, *Serratula coronata* L., *S. quinquefolia* M. Bieb. ex Willd. (Figure S1) and others.

From the aerial parts of the above-mentioned plants, sesquiterpene lactones, coumarins, phytoecdysones and phenol compounds have been isolated. The isolation of the active compounds was conducted based on the procedures specified for the sesquiterpene lactones [12], phytoecdysones [8], coumarins [13] and phenolic glycosides [14].

Isolated compounds were identified by NMR spectra. They were run on a Bruker Avance 600 instrument using 600 and 150 MHz frequencies for hydrogen nuclei (^1^H) and carbon nuclei (^13^C), respectively, and tetramethylsilane (TMS) was used as an internal standard. The spectra were obtained for CDCl_3_ or DMSO-*d_6_* solutions at 298 K. Chemical shifts are given in ppm, and coupling constants *J* are given in Hz (Tables S1–S3; Figure S2) [15,16]. Absolute configuration of the dominant compound from the *S. quinquefolia* leaf was based on the crystallography method (Figure S3) [17].

In our studies, over twenty new compounds were isolated and identified. For the last ten years, the focus of our study has been on pharmacological properties of isolated compounds and the possibilities of creating new drugs with the extracts from Centaureinae plants as active substances. Table 1 shows the pharmacological properties of selected extracts and compounds from some plants of the Centaureinae subtribe.

As mentioned above, compounds from Centaureinae plants have differentiated pharmacological properties, which is why it is very important to choose the appropriate biological study for specific compounds or plant extracts. Therefore, the chemical structure of compounds was also carefully analyzed, making it possible to establish a correlation between compounds’ chemical structure and their biological properties.

Based on previous studies and on compounds’ chemical structure, we have decided to test the compounds and plant extracts in treating antifungal infections, seborrheic dermatitis and hyperpigmentation. We have also conducted tests for serotonin inhibition properties, which may help in finding new ways of treating migraines.

The aim of this review was to describe recent studies in the field of phytotherapy and present the results concerning the pharmacological properties of compounds from plants of the Centaureinae subtribe, which may help in future treatment of antifungal infections, migraine, seborrheic dermatitis and hyperpigmentations.

## 2. Selected Centaureinae (Asteraceae) Plant Materials in Phytotherapy

### 2.1. Phytotherapy Possibilities for Treating Fungal Infections

Fungal infections affect about 40% of the world′s population and may be viewed as an epidemiological, therapeutic and social problem. Over a billion people are directly affected by mycoses globally, 150 million of whom have a serious or life-threatening infection [42]. Fungal infections can cause serious illnesses, several of which may be fatal if left untreated. Commonly used antibiotics change human microflora and consequently increase the number of people with impaired immunity [43].

Such factors as the development of industry, agriculture, technology and life extension make the population more susceptible to infections [44]. The number and variety of fungi causing infections are increasing all over the world. Not only in different parts of the world but sometimes even within one country, there are differences in fungal flora, and species of fungi can be identified with variable frequency of occurrence. Among most commonly occurring fungi infections are *aspergillosis*, *coccidioidomycosis*, *candidosis*, *cryptococcosis*, *mycetomas*, *histoplasmosis*, *mucormycosis*, and *paracoccidio-idomycosis* [45].

Conventional antifungal treatment is based on polyene agents, flucitosine and azole agents, or more recently, on virulence factor inhibitors and immunomodulators. This has led to the production of new and improved azoles and polyene formulations, as well as a new family of drugs, the echinocandins [46].

Some germacranolides isolated from *Centaurea* species showed antifungal activity against *Cunninghamella echinulate*. The authors who found this concluded that a relatively low polarity is one of the molecular requirements for the antifungal activity of sesquiterpene lactones [47].

Coumarins are compounds with lactone structure that also show antifungal activity. Some coumarin derivatives were tested against the fungal strains *Candida albicans* (ATCC 14053), *Aspergillus fumigatus* (ATCC 16913) and *Fusarium solani* (ATCC 36031) using the broth microdilution method and showed strong antifungal activity [48]. Coumarins also exhibit anti-inflammatory activity [49].

Furthermore, 0.39% guaianolides **1**, **11**, **12**, **13**; the mixture of guaianolides **1**–**4**; a coumarin compound **29** and dry methanol extracts from *P. bellus* herb with 26 sesquiterpene lactones (compounds **1**–**26**); and *P. sibiricus* leaf with four coumarins (compounds **27**–**30**) (Table 1) were used for an antifungal study against following clinical strains of fungi: *Candida albicans*, *C. famata*, *C. glabrata*, *C. parapsilosis*, *Rhodotorula rubra*, *Trichophyton rubrum*, *T. mentagrophytes var. interdigitale*, *Microsporum canis* and *Scopulariopsis brevicaulis.*

Pathogenic fungi were collected from the patients with fungal infection diagnosed by a dermatologist. The patients were not treated with any other antifungal drugs.

Clinical strains of fungi were suspended in 0.9% NaCl solution and adjusted to the desired concentration of 1 in McFarland standard. The suspension was transferred to the sterile paper discs and placed in the Petri dishes. After drying for about 15 min, the surface was spread with 10 μg of the studied substance dissolved in dimethylsulfoxide (DMSO) (concentration 0.39%). After 15 min, the Petri dishes were incubated. *Candida* cultures were incubated for 72 h at 36 °C. Dermatophytes (*Trichophyton* sp., *Rhodotorula* sp., *Microsporum* sp.) and mold fungus (*Scopulariopsis brevicaulis*) were incubated at 27 °C for two weeks (Figure S4).

The inhibition zones’ diameter (in mm) was measured, and thus the antifungal properties of the studied substances were assessed. Fungal growth analysis was performed under a stereomicroscope (Nikon SMZ800, Tokyo, Japan) at the Microbiology Section (Department of Dermatology, Poznan University of Medical Sciences, Poznan, Poland).

The analysis of all fungi cultures suitable for assessment showed that all of the studied compounds have antifungal properties (Table 2). Due to some compounds′ limited amounts, only 0.39% solutions of the compounds and extracts were used during the study.

The diameters of the inhibition zones were measured to assess how susceptible the fungi are to the activity of the analyzed compounds. The analyzed fungi could be classified into the following categories:

Very Susceptible, with the diameter of inhibition zone 20 mm or over 20 mm
Susceptible: with the diameter of inhibition zone between 10 mm and 19 mm
Moderately susceptible: with the diameter of inhibition zone between 1 mm and 9 mm
Resistant: no inhibition zone

The measurements were taken for three days from *Candida* cultures and 14 days from the dermatophytes and mold fungus (*Scopulariopsis brevicaulis*). The assessment of compounds′ antifungal activity on *Candida albicans* was only possible in the case of cebellins **2**–**4** mixture and cebellin A (**11**) due to the difficulties with growing these fungi strain cultures. Other strains turned out to be easier to grow, which is why the antifungal activity of all of the studied compounds could have been observed [50].

**Figure 1:**
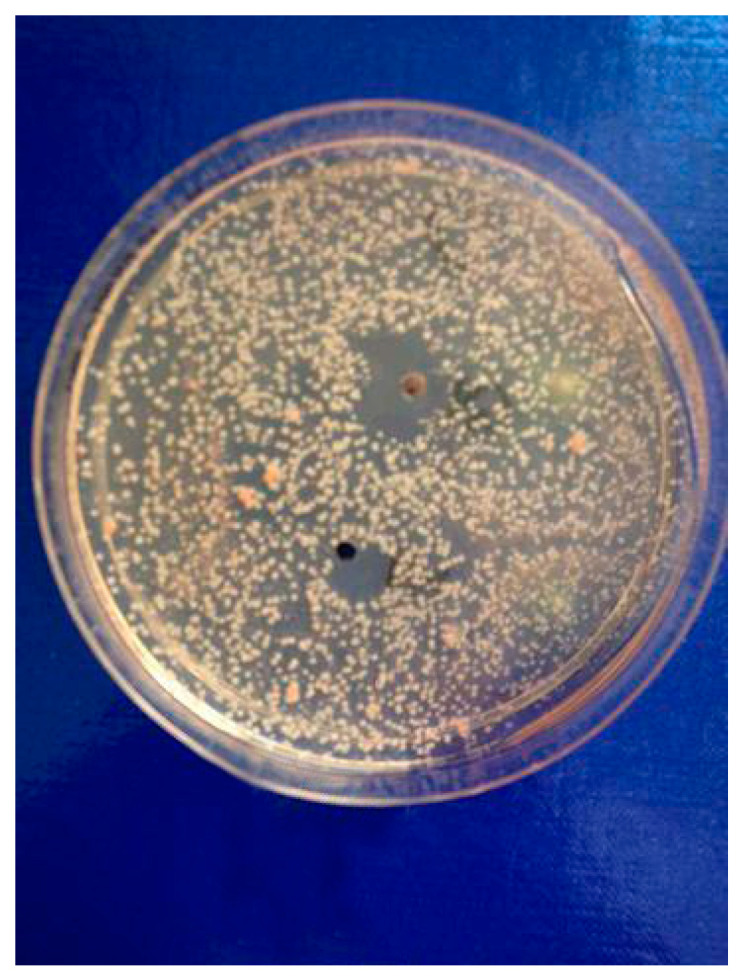
The results of susceptibility assessment of *Rhodotorula rubra* to the mixture of C-2 ester guaianolides (compound **1** inhibition zone diameter = 12, compounds **2**–**4** inhibition zone diameter = 22 mm).

The most significant number of fungi (among them *Candida albicans, Microsporum canis* and *Rhodotorula rubra*) strains turned out to be very susceptible to cebellins **1**–**4** from the *P. bellus* herb (Figure 1). These compounds are the most lipophilic of the studied lactones. [47].

Yeast-like fungi *Candida famata* and *C. glabrata,* as well as dermatophytes from the *Trichophyton* genus, *T.* *rubrum* and *T. mentagrophytes var. interdigitale,* were the most susceptible to the analyzed compounds. The highest potency of the *P. bellus* herb extract was shown (inhibition zone′s diameter reached 34 mm) [50].

It may be suggested that low-polar sesquiterpene lactones (compounds **1**–**13**), mostly those with an ester on C2 (compounds **1**–**4**, **11**, **12**) are responsible for such a strong antifungal effect of the extract from the *P. bellus* herb.

Scopoletin (**29**) (yielded in yellow crystals—Figure S2) also showed antifungal properties, and the strains *Candida famata*, *C. glabrata* and *Trichophyton rubrum* were susceptible to it. On the other hand, *T. mentagrophytes var. interdigitale* and *Scopulariopsis brevicaulis* turned out to be only moderately susceptible to this compound.

Six guaianolides isolated from the *P. bellus* herb (**1**–**4**, **11**, **12**) have an additional ester on C2. Its structure (methyl groups) and location might be related to the compound’s lipophilicity. The compounds with the additional ester have higher R_f_ value in a nonpolar mobile phase, which can be interpreted as a lipophilicity indicator (Figure S5). Further research is needed to establish whether there is a correlation between the presence of this particular ester in the compounds’ chemical structure and their lipophilicity, which suggests the compounds′ ability to penetrate through the fungal cell wall and consequently destroying it [47].

In the chemical structure of each of the isolated coumarins from the leaves of *P. sibiricus*, a lactone ring can be observed. As the lactones have proven anti-microbial properties, this element of the structure was a reason for choosing these compounds for biological studies. The second reason was the coumarins’ lipophilicity, as shown on the chromatogram (Figure S6). The coumarins have a high R_f_ value in a nonpolar mobile phase.

### 2.2. Possibilities for Phytotherapy in Serotonin Inhibition

Chronic migraine headaches are an important health problem. A headache in a migraine episode is described as hemicranial, pulsating and so intense that it strongly interferes with the patient’s everyday life [51]. One explanation of the origin of migraines is the “serotonin theory”, confirmed by the increased excretion of serotonin metabolites in urine during the headache episode. Serotonin (5-HT) is released from platelets, which causes contraction of the smooth muscle of the blood vessels [52]. Afterward, as a result of biochemical changes, the serotonin level decreases, causing vasodilation and an increase in vascular permeability, allowing the flow of substances able to lower the sensitivity threshold of perivascular space nociceptor [53].

Parthenolide (**31**) is a sesquiterpene lactone derived from the leaves of Feverfew (*Tanacetum parthenium*) and is considered the main bioactive component of this herb [54]. Feverfew is used orally or as an infusion for the treatment of migraine, arthritis, fever and stomachache [55]. Parthenolide (**31**) reduces the cellular level of GSH in cancer cells, followed by ROS accumulation and apoptosis [56]. Parthenolide’s ability to induce cell death, mainly in cancer cells, while sparing healthy cells is unique and may be linked to the presence of 4,5-epoxide, lactone ring and an exo-methylene [57]. The compound also protects normal cells from UVB and oxidative stress, and it seems to have the potential to target some cancer stem cells [58].

The serotonin inhibition ex vivo study covered germacranolides **33**, **34**, **38** and 2% extract from the *St. balsamita* leaf with seven parthenolide (**31**) derivatives (compounds **32**–**38**). During the study, it was proven that izospiciformin (**33**), stizolin (**34**) and stizolicin (**38**), as well as the extract with four additional germacranolides (**32, 35**–**37**), inhibit the release of 5-HT from platelets more effectively than parthenolide (**31**), and the results were statistically significant: (**31** *p* = 0.0477; **33** *p* = 0.0001; **34** *p* = 0.0380; **38** *p* = 0.0389; methanol extract from *St. balsamita* leaf *p* = 0.0097) [59]. Izospiciformin (**33**) and the ethanol extract from the *St. balsamita* leaf showed the most potent effect.

Two elements may be found in the chemical structures of the guaianolides isolated from *P. bellus* herb as well as in germacranolides isolated from the *St. balsamita* leaf. A lactone ring coupled with an exo-methylene enables inhibition of the cellular enzymes through Michael nucleophilic addition [60]. In consequence, these compounds exhibit antiviral activity, including that of the SARS-CoV-2 virus [61], as well as antiprotozoal [62] and antiserotonin activity, the latter being responsible for lactones’ antimigraine effect [63].

In *St. balsamita* leaf, there are seven parthenolide derivatives (**32**–**38**), all of which contain 4,5-epoxide. The difference in the chemical structure between the parthenolide (**31**) and its derivatives from *St. balsamita* should be stressed. Compounds **32**–**38**, along with the three above-mentioned elements characteristic for a parthenolide (4,5-epoxide, lactone ring and an exo-methylene), have an additional element, namely a substituent on C-8, which significantly increases the potency of the antiserotonin effect compared to parthenolide [59].

### 2.3. Phytotherapy’s Possibilities in Treating Seborrheic Dermatitis

The bothersome symptoms of seborrheic dermatitis (SD) are difficult to control. SD is a chronic dermatitis characterized by erythema and skin flaking, which occur most often on the face, scalp, ears, chest and body folds—in other words, places with a high concentration of sebaceous glands [64].

20-hydroxyecdysone derivatives found in plants of the *Serratula* genus, when applied to the skin, restore dermis and strengthen protective functions of the epidermis, making the skin more hydrated and resilient. Phytoecdysones may therefore be used in dry and very dry skin care and eases such symptoms as ichtyosis and psoriatic conditions [65]. Phytoecdysones are able to activate keratinocytes and increase their amount and differentiation, which is why those compounds are used in projects of creating artificial skin [66].

It is postulated that phytoecdysones may have a significant impact on the reduction of inflammation, probably through their immunomodulatory function and the modulation of proinflammatory cytokines level (e.g., IL-6, TNF-α) [67]. Moreover, it has been reported that phytoecdysteroids improve skin quality by accelerating the healing process of wounds and burns [68]. Several studies [69] have claimed associations between *Malassezia restricta* lipase and seborrheic dermatitis. In light of this, ecdysteroids, especially 20-hydroxyecdysone (**41**), probably enhance antifungal immunity, resulting in the reduction of disease symptoms [70].

Ecdysteroids from plants of the Centaureinae subtribe are characterized by two OH groups on C2 and C3. Some, though only those of vegetable origin, have extra hydroxyl groups on C1, C5 and C11 (Table 1). The presence of those additional groups seems to translate into increased safety in use [71].

*S. coronata* herb is the source of phytoecdysones [58], which were studied for their use in easing the symptoms of seborrheic dermatitis. Dry ethanol extract from *S. coronata* herb standardized in its content of three dominating phytoecdysones—ajugasterone C (**39**), polypodine B (**40**) and 20-hydroxyecdysone (**41**)—was used as an active substance in preparing the cream. The amphiphilic cream base was chosen specifically for its ability to easily release the active substance (Lekobaza Pharma Cosmetic, Fargon, Poland). The base was mixed with water extract containing 22.39% ecdysones to achieve a 2.5% concentration of the active substance in the cream. Each patient was treated with 8 mg (=1.8 mg of the active substance) of the cream, which was applied, directly to the changed skin, two times a week for six weeks [59].

The cream with phytoecdysones (compounds **39**–**41**) proved to be an effective and safe preparation for treating skin changes caused by seborrheic dermatitis [59].

### 2.4. Phytotherapy’s Possibilities in Treating Skin Discoloration

Skin hyperpigmentation is a common cause of patients’ visits to beauty shops and dermatological clinics. Usually located on the exposed parts of the body (face, neck, neckline, forearms and backs of the hands), these changes may be the result of inflammation, endocrine disorders and systemic diseases, as well as UV radiation, phototoxic or photoallergic substances contained in medicines, herbs and cosmetics. They occur due to the disturbance of melanin synthesis and abnormal distribution of melanin in the skin [72].

Lighter skin tones have long been associated with youth and beauty among a variety of Asian cultures. Investment in skin-whitening agents, boosted by markets in Asian countries, especially those in China, India and Japan, is increasing annually. Skin color is influenced by a number of intrinsic factors, including skin types and genetic background, and extrinsic factors, including the degree of sunlight exposure and environmental pollution [73].

Cosmeceuticals are commonly used for hyperpigmentation. These disorders are generally difficult to treat, hence the need for skin-lightening agents. Arbutin in cream is used as a first choice in treating hyperpigmentation [74].

β-arbutin, phenol glucoside, is a compound with anti-inflammatory effect. Moreover, its mechanism of action is based on inhibiting the activity of tyrosinase, a vital enzyme in the process of melanin synthesis [75]. As it was possible to isolate an unexpectedly large quantity of β-arbutin (**42**) from *St. quinquefolia* plant material without methylarbutin and hydroquinone (Figure S7) [14], water extract from the leaves of *S. quinquefolia* was used for biological studies. The extract was tested during the clinical trial for their efficiency in skin discoloration, specifically melasma and lentigo solaris treatment. A cream containing 2.51% of active compound was applied to the discolored place twice a day—in the morning and the evening (one application of 100 mg = 2.5 mg of the active substance)—for eight weeks [76].

The cream with this extract decreased melanin level in the skin pigmentation spots. The clinical effect, in the form of lightening and evening skin tone on the discolored side, was observed in 75.86% of the female patients with melasma and 56.00% of the female patients with lentigo solaris. The cream with the aqueous extract from the leaf of five-leaf *Serratula* proved to be an effective and safe preparation for lightening skin discoloration (66.67% of the female patients in the study group) [76].

## 3. Conclusions

Recent studies show that searching for new possibilities of phytotherapy using compounds isolated from Centaureinae plants is worth the effort. Treatment based on active substances from plants of the Centaureinae subtribe is often effective and does not cause side effects, as was demonstrated on an example of antifungal infections and SD and melasma and lentigo solaris treatment.

There seem to be a correlation between the chemical structure of compounds and their pharmacological properties, which may be helpful, e.g., in selecting the right biological studies for specific compounds. Further research is needed on this issue.

## Figures and Tables

**Table 1:** Pharmacological properties of the compounds and the extracts from selected plants of Centaureinae subtribe.

Source	Compound	Structure	Properties/Uses
*Psephellus bellus* herb	Cebellin L (**1**) Budesinsky et al. (1994) [15]	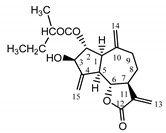	anti-inflammatory/antifungal
	Cebellin O (**2**) Daniewski and Nowak (1993) [16]	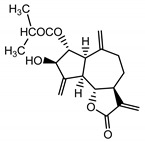	anti-inflammatory/antifungal
	Cebellin K (**3**) Budesinsky et al. (1994) [15]	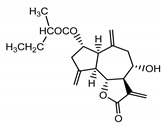	anti-inflammatory/antifungal
	Cebellin N (**4**) Daniewski and Nowak (1993) [16]	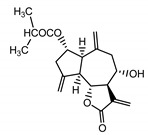	anti-inflammatory/antifungal
	19-deoxychloro-janerin (**5**) El-Dahmy et al. (1985) [18]	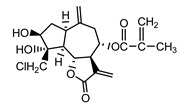	anti-inflammatory/antifungal (in extract)
	17,18-epoxy-19-deoxy-chlorojanerin (**6**) Budesinsky et al. (1994) [16]	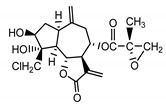	anti-inflammatory/antifungal (in extract)
	Cebellin M (**7**) Budesinsky et al. (1994) [16]	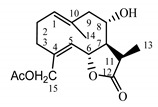	anti-inflammatory/antifungal (in extract)
	8-desacylo-8α-(2′-methyl-acryloxy)-subluteolide (**8**) Bohlmann and Ziesche (1980) [19]	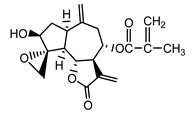	anti-inflammatory/antifungal (in extract)
	Repin (**9**) Gonzales et al. (1977) [20]	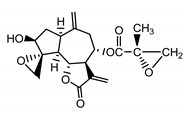	anti-inflammatory/antifungal (in extract)
	Centaurepensin (**10**) Gonzales et al. (1974) [21]	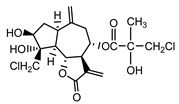	anti-inflammatory/antifungal (in extract)
	Cebellin A (**11**) Nowak et al. (1986a) [22]	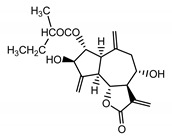	anti-inflammatory/antifungal
	Cebellin B (**12**) Nowak et al. (1986a) [22]	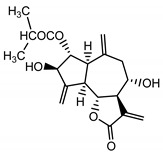	anti-inflammatory/antifungal
	Acroptilin (**13**) Estratova et al. (1967) [23]	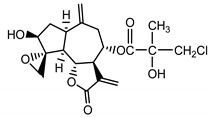	anti-inflammatory/antifungal (in extract)
	Cynaropicrin (**14**) Samek et al. (1971) [24]	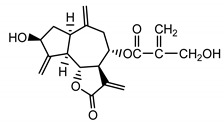	anti-inflammatory/antifungal (in extract)
	Cebellin F (**15**) Nowak et al. (1986a) [22]	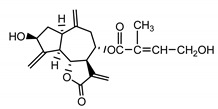	anti-inflammatory/antifungal (in extract)
	15-deoxyrepin (**16**) Nowak et al. (1986b) [25]	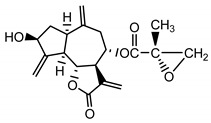	anti-inflammatory/antifungal (in extract)
	Chlorojanerin (**17**) Stevens (1982) [26]	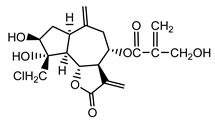	anti-inflammatory/antifungal (in extract)
	8-desacetyl-centaurepensin-8-O-(4′-hydroxy)-tiglate (**18**) Stevens (1982) [26]	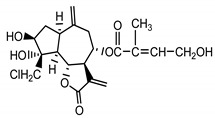	anti-inflammatory/antifungal (in extract)
	Repensolide (**19**) Jakupovic et al. (1986) [27]	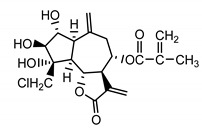	anti-inflammatory/antifungal (in extract)
	Janerin (**20**) Gonzales et al. (1977) [20]	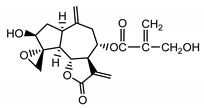	anti-inflamatory/antifungal (in extract)
	8-4′-tiglinate-8-desacetyl-subluteolide (**21**) Budesinsky et al. (1994) [16]	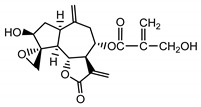	anti-inflamatory/antifungal (in extract)
	Cebellin G (**22**) Nowak et al. (1986a) [22]	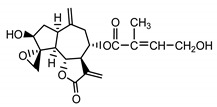	anti-inflamatory/antifungal (in extract)
	Cebellin H (**23**) Nowak et al. (1986a) [22]	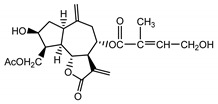	anti-inflamatory/antifungal (in extract)
	Cebellin I (**24**) Nowak et al. (1986a) [22]	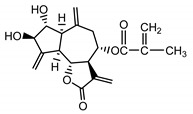	anti-inflammatory/antifungal (in extract)
	Repdiolide (**25**) Bohlmann et al. (1982) [28]	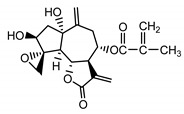	anti-inflammatory/antifungal (in extract)
	Cebellin J (**26**) Budesinky et al. (1994) [16]	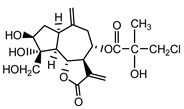	anti-inflammatory/antifungal (in extract)
*Psephellus sibiricus* leaf	Coumarin (**27**) Dean (1952) [29]	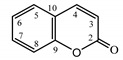	anti-inflammatory/antifungal (in extract)
	Scoparone (**28**) Ma et al. (2006) [30]	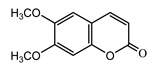	anti-inflammatory/antifungal (in extract)
	Scopoletin (**29**) Tsukamoto et al. (1984) [31]	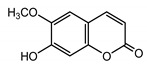	anti-inflammatory/antifungal
	Umbelliferone (**30**) Hulting (1967) [32]	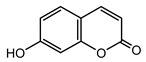	anti-inflammatory/antifungal (in extract)
*Tanacetum parthenium* herb	Parthenolide (**31**) Hevlett et al. (1996) [33]	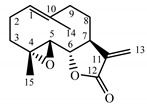	anti-inflammatory/antimigraine/ Anticancer
*Stizolophus balsamita* leaf	Balsamin (**32**) Rybalko et al. (1969) [34]	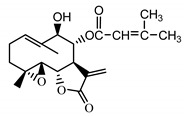	anti-inflammatory/antiserotonin (in extract)
	Izospiciformin (**33**) Nowak et al. (1989) [35]	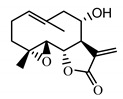	anti-inflammatory/antiserotonin (in extract)
	Stizolin (**34**) Mukametzhnov et al. (1971) [36]	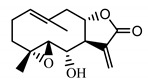	anti-inflammatory/antiserotonin (in extract)
	9α-hydroxy-parthenolide (**35**) Tyson et al. (1981) [37]	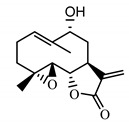	anti-inflammatory/antiserotonin (in extract)
	8-*E*-(4′-hydrohy)-senecioyloxy-9α-hydroxyparthe-nolide (**36**) Oksuz and Ayyildiz (1986) [38]	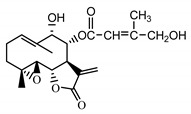	anti-inflammatory/antiserotonin (in extract)
	11βH,13-dihydro-stizolicin (**37**) Nawrot et al. (2019) [4]	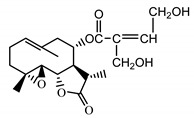	anti-inflammatory/antiserotonin (in extract)
	Stizolicin (**38**) Mukametzhanov et al. (1971) [36]	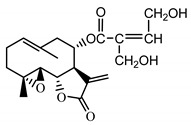	anti-inflammatory/antiserotonin
*Serratula coronata* herb	Ajugasterone C (**39**) Imai et al. (1969) [39]	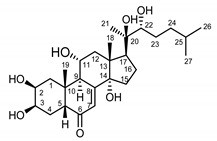	anti-*Malassesia restricta* Seborrheic dermatitis (in extract)
	Polypodine B (**40**) Jizba et al. (1967) [40]	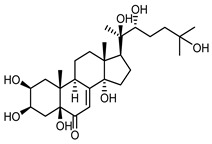	anti-*Malassesia restricta* Seborrheic dermatitis (in extract)
	20-hydroxyecdysone (**41**) Hocks and Wiechert (1996) [41]	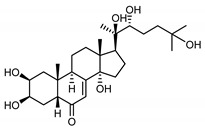	anti-*Malassesia restricta* Seborrheic dermatitis (in extract)
*Serratula quinquefolia* leaf	β-arbutin (**42**) Nycz et al. (2010) [17]	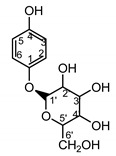	hyperpigmentations (in extract)

**Table 2:** The assessment of fungi susceptibility to studied natural compounds.

Herbal Substance	*Candida albicans*	*Candida famata*	*Candida glabrata*	*Candida parapsilosis*	*Rhodotorula rubra*	*Trichophyton rubrum*	*Trichophyton mentagrophytes var. interdigitale*	*Microsporum canis*	*Scopulariopsis brevicaulis*
Cebellin L	-	S	S	VS	S	S	VS	-	S
Cebellins K + N + O	S	-	VS	-	VS	-	VS	VS	MS
Cebellin A	M	-	S	-	S	-	S	-	VS
Cebellin B	-	S	S	MS	-	S	S	-	MS
Acroptilin	-	MS	-	-	-	VS	MS	-	-
Scopoletin	-	S	S	MS	-	S	MS	-	MS
*P. bellus* extract	-	S	S	S	-	VS	VS	-	-
*P. sibiricus* extract	-	S	-	S	-	-	S	-	-

- the measurements could not be taken. VS = Very susceptible, with the diameter of inhibition zone over 19 mm. S = Susceptible, with the inhibition zone between 10 mm and 19 mm. MS = Moderately Susceptible, with the diameter of inhibition zone between 1 mm and 9 mm. R = Resistant: no inhibition zone.

## Supplementary Materials

The Supplementary Materials are available online—Figure S1: Plants in the Garden of Medicinal Plants in the Department and Division of Practical Cosmetology and Skin Diseases Prophylaxis, Poznan University of Medicinal Sciences; Table S1: ^1^H NMR (600 MHz) spectroscopic data (δ_H_ in ppm, mult; *J* in Hz) of compounds: **1**, **11**–**13**; Table S2: ^1^H NMR (600 MHz) spectroscopic data (δ_H_ in ppm, mult; *J* in Hz) of: izospiciformin (**33**), stizolin (**34**), and stizolicin (**38**); Table S3: ^1^H NMR data (600,20 MHz) of ajugasterone C (**39**), polypodine B (**40**) and 20-hydroxyecdysone (**41**) (in CD_3_OD); Figure S2: ^1^H NMR spectroscopic data of compound **29** and crystals of scopoletin; Figure S3: X-ray analysis of β-arbutin from *S. quinquefolia* leaf and crystals of arbutin; Figure S4: *Candida glabrata*, *Trichophyton rubrum*, *Microsporum canis*, *Scopulariopsis brevicaulis* cultures; Figure S5: TLC of compounds from *Psephellus bellus* herb; Figure S6: TLC of coumarins from *Psephellus sibiricus* leaf; Figure S7: The HPLC chromatogram of the water extract from the *S. quinquefolia* leaf.
