# Supplementary material for: Phytotherapy Perspectives for Treating Fungal Infections, Migraine, Sebhorreic Dermatitis and Hyperpigmentations with the Plants of the Centaureinae Subtribe (Asteraceae)
Source: Molecules. 2020 Nov 15;25(22):5329. doi: 10.3390/molecules25225329 (PMC7696306; doi:10.3390/molecules25225329)
Supplement: Supplementary file 1 [file molecules-25-05329-s001.pdf]

Supplementary Materials

Phytotherapy Perspectives for Treating Fungal Infections, Migraine, Sebhorreic Dermatitis and Hyperpigmentations with the Plants of the Centaureinae Subtribe (Asteraceae)

Joanna Nawrot, Justyna Gornowicz-Porowska and Gerard Nowak *

Department and Division of Practical Cosmetology and Skin Diseases Prophylaxis, Poznan University of Medical Sciences, 33 Mazowiecka Street, 60-623 Poznań, Poland; joannac@ump.edu.pl (J.N.); justynagornowicz1@poczta.onet.pl (J.G.-P.)

* Correspondence: gnowak@ump.edu.pl; Tel./Fax: +48-61-8470628

Figure S1. Plants in the Garden of Department and Division of Practical Cosmetology and Skin Diseases Prophylaxis, Poznan University of Medicinal Sciences.
Table S1. ^{1}H NMR (600 MHz) spectroscopic data (δ_H in ppm, mult; *J* in Hz) of compounds: **1**, **11**-**13** isolated from *P. bellus* herb
Table S2. ^{1}H NMR (600 MHz) spectroscopic data (δ_H in ppm, mult; *J* in Hz) of: izospiciformin (**33**), stizolin (**34**), and stizolicin (**38**)
Table S3. ^{1}H NMR data (600,20 MHz) of ajugasterone C (**39**), polypodine B (**40**) and 20- hydroxyecdysone (**41**) (in CD_3OD)
Figure S2. ^{1}H NMR spectroscopic data of compound **29** and crystals of scopoletin.
Figure S3. X-ray analysis of β-arbutin from *S. quinquefolia* leaf and crystals of arbutin,
Figure S4. *Candida glabrata, Trichophyton rubrum, Microsporum canis, Scopulariopsis brevicaulis* cultures.
Figure S5. TLC of lipophilic compounds from *Psephellus bellus* herb; Mobile phase: hexane – CH_2Cl_2 – AcOEt 4:2:5.
Figure S6. TLC of coumarins from *Psephellus sibiricus* leaf
Figure S7. The HPLC chromatogram of the water extract from the *S. quinquefolia* leaf.

Figure S1. Studied plants in the Garden of Department and Division of Practical Cosmetology and Skin Diseases Prophylaxis, Poznan University of medicinal Sciences

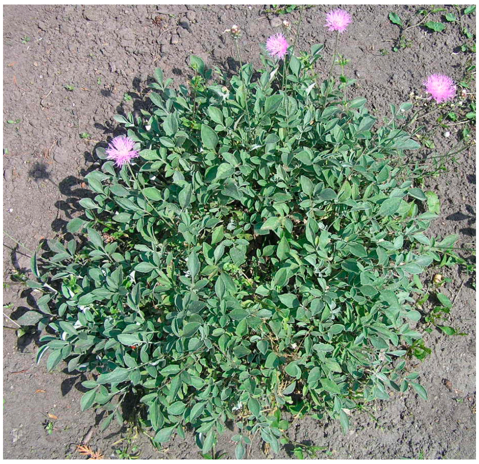

Psephellus bellus (*Centaurea bella*)

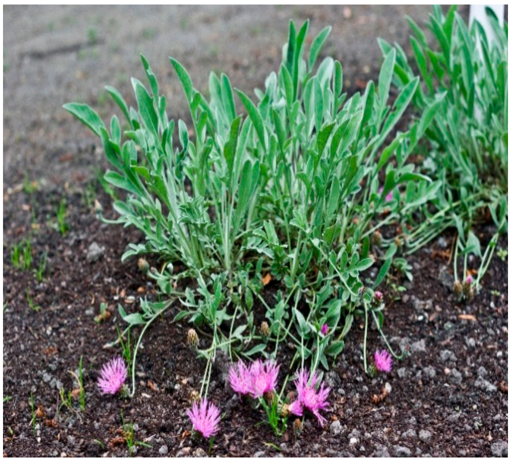

Psephellus sibiricus (*Centaurea sibirica*)

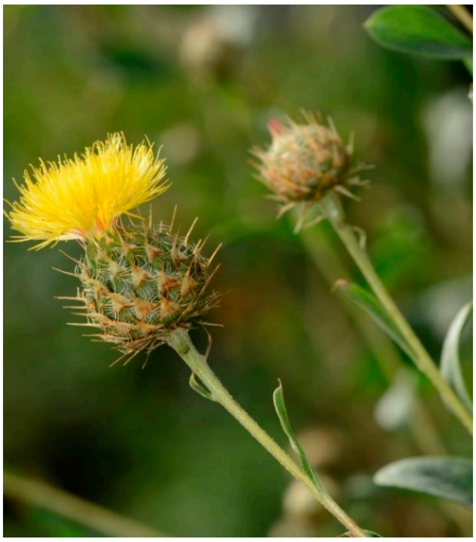

Stizolophus balsamita

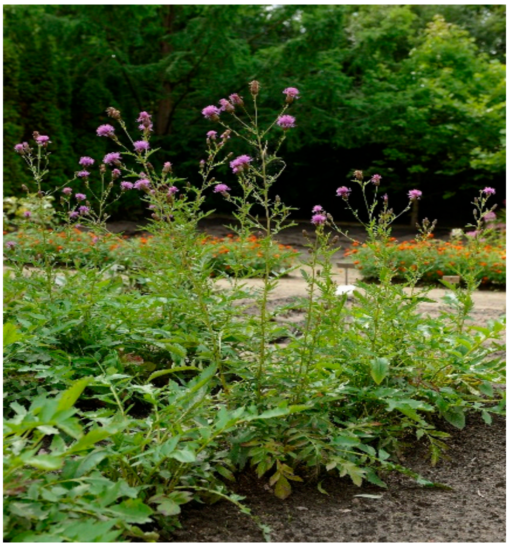

Serratula coronata

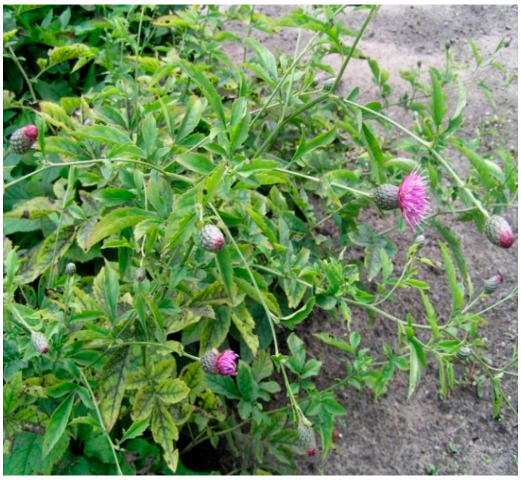

Serratula quinquefolia

Table S1. ^{1}H NMR (600 MHz) spectroscopic data (δ$_H$ in ppm, mult; *J* in Hz) of compounds: **1**, **11**-**13** from *P. bellus* herb

Pos.	**1** [a]	**11** [a]	**12**[a]	**13**[b]
1	2.93 dd (6.5, 9.8)	2.98 m	2,98 m	3.38 m
2	-	-	-	2.51 m
2	4.94 dd (6.5; 5.8)	4.98 dd (9.3; 7.3)	4.96 dd (9.0; 7.2)	1.81 m
3	4.41 m	4.43 m	4.43 m	3.99 m
5	3.01 t	2.98 m	2.85 m	2.02 d (11.3)
6	4.16 dd (9.0; 9.8)	4.16 dd (10.6; 9.0)	4.25 dd (10.1; 9.0)	4.08 d 9.3)
7	2.88 m	2.81 m	3.19 m	3.08 t (9.4; 3.5; 3.2
8	2.25 m	4.01 m	4.01 dd (3.0; 5.3)	5.24 m
8	2.24 m	-	-	-
9	2.25 m	2.32 dd (5.4; 14.6)	2.37 dd (5.3; 14.6)	2.49 dd (15.2; 2.7)
9	2.47 m	2.70 dd (14.6; 5.4)	2.70 dd (14.6; 5.3)	2.71 dd (15.2; 5.2)
13a	6.25 d (3.6)	6.29 dd (3.5. 0.8)	6.21 d (0.8)	6.24 d (1.6)
13b	5.52 d (3.2)	6.16 dd (3.2; 0.8)	6.16 d (0.8)	5.57 d (1.6)
14a	4.98 d (2.5)	5.11 d (1.6)	5.14 d (1.7)	5.21 d (1.8)
14b	4.93 d (2.1)	5.03 d (1.6)	4.92 d (1.7)	5.11 d (1.8)
15a	5.52 d (2.1)	5.66 dd (1.5; 0.8)	5.66 dd (0)	3.34 d (4.2)
15b	5.44 d (1.8)	5.47 dd (1.7; .8)	5.47 m	3.07 d (4.2)
8-OH		1.85 brs	1.85 brs	
2′	2.40 m	2.42 m	2.48 m	-
3′	1.67 m; 1.48 m	1.63 m; 1.51 m	1.17 d	3.88 d
4′	0.90 m	1.15 d	1.16 d	1.55 s
5′	1.16 q	0.69 m	-	-

Table S2. ^{1}H NMR (600 MHz) spectroscopic data (δ_H in ppm, mult; *J* in Hz) of: izospiciformin (**33**), stizolin (**34**), and stizolicin (**38**) from *St balsamita* leaf.

Pos.	**33** [a]	**34** [a]	**38** [b]
1	5.36 bs	5.25 bd (12.1)	5.45 bd (10.5)
2a	1.21 m	2.43 m	2.52 dd (5.8;13.5)
2b	0.98 m	2.23 m	2.25 bd (13.6)
3a	2.13 m	2.15 m	2.13 dd (12.9; 6.0)
3b	1.34 m	1.24 m	1.29 m
5	2.66 d (8.9)	2.73 d (8.1)	2.85 d (9.3)
6	3.44 dd (8.9;9.6)	3.98 bt (7.4)	4.37 t (9.3)
7	3.00 m	3.09 m	3.59 m
8a	4.01 m	-	4.57 bdd (3.9; 2.7)
8b	-	3.88 m	-
9a	2.84 m	2.57b d (12.5)	2.70 dd (11.8; 11.6)
9b	2.14 m	2.43 d (12.2)	2.47 bd (12.1)
13a	6.46 dd (1.2; 3.0)	6.50 d (3.1)	5.80 d (3.0)
13b	6.28 dd (1.2; 2.6)	6.16 d (2.4)	6.20 d (3.4)
14	1.79 s	1.76 s	1.82 bs
15	1.28 s	1.28s	1.28 bs
3’	-	-	6.94 t (5.9)
4’	-	-	4.40 d (5.9)
5’	-	-	4.28 s

Table S3. ^{1}H NMR data (600,20 MHz) of ajugasterone C (**39**), polypodine B (**40**) and 20-hydroxyecdysone (**41**) (in CD_3OD) from *S. coronata* herb

Proton	**39** δ_H (ppm) (*J* Hz)	**40** δ_H (ppm) (*J* Hz)	**41** δ_H (ppm) (*J* Hz)
1a	2.58 dd (12.9; 4.0)	1.68 m	1.43 m
1b	1.38 m	1.78 m	1.78 dd (4.6; 13.3)
2	4.01 dt (4.0)	3.94 h (3.5; 6.5; 10.1)	3.83 tt (3.8; 8.2; 8.0)
3	3.95 m	3.99 bq (3.0; 6.2; 9.18)	3.94 bd (2.1)
4a	1.78 m	1.75 m	1.65 m
4b	1.69 m	2.07 dd (2.9; 14.8)	1.75 m
5	2.33 dd (3.7; 13.15)	-	2.38 dd (3.9; 10.0)
7	5.80 d (2.0)	5.85 d (2.7)	5.80 d (2.5)
9	3.15 m	3.19 m	3.14 dd (4.93)
11a	4.10 m (13.3)	1.72 m	1.65 m
11b	-	1.81 m	1.78 m
12a	2.21 m	2.13 m (4.9; 13.0)	2.13 ddd (4.8; 13.0;13.0)
12b	2.15 dd (5.9; 12.1)	1.88 m	-
15a	1.97 m	1.59 m	2.00 m
15b	1.56 m	-	1.55 m
16a	1.70 m	2.00 m	1.95 m
16b	1.99 m	1.74 m	1.75 m
17	2.41 m	2.39 m	2.39 m (3.9)
18	0.87 s	0.89 s	0.89 s
19	1.05 s	0.92 s	0.96 s
21	1.19 s	1.192 s	1.187 s
22	3.30 m	1.76 bm	3.33 d (1.5)
23a	1.54 m	1.28 m	1.30 m
23b	1.20 m	1.67 m	1.65 m
24a	1.47 m	1.77 m	1.75 m
24b	1.23 m	1.44 m	1.45 m
25	1.58 m	-	-
26	0.916 d (6.2)	1.187 s	1.195 s
27	0.920 d (6.2)	1.200 s	1.200 s

Figure S2. ^{1}H NMR (600 MHz, DMSO-*d*$_6$) spectrum of compound **29.** SCOPOLETIN from *P. sibiricus* leaf.

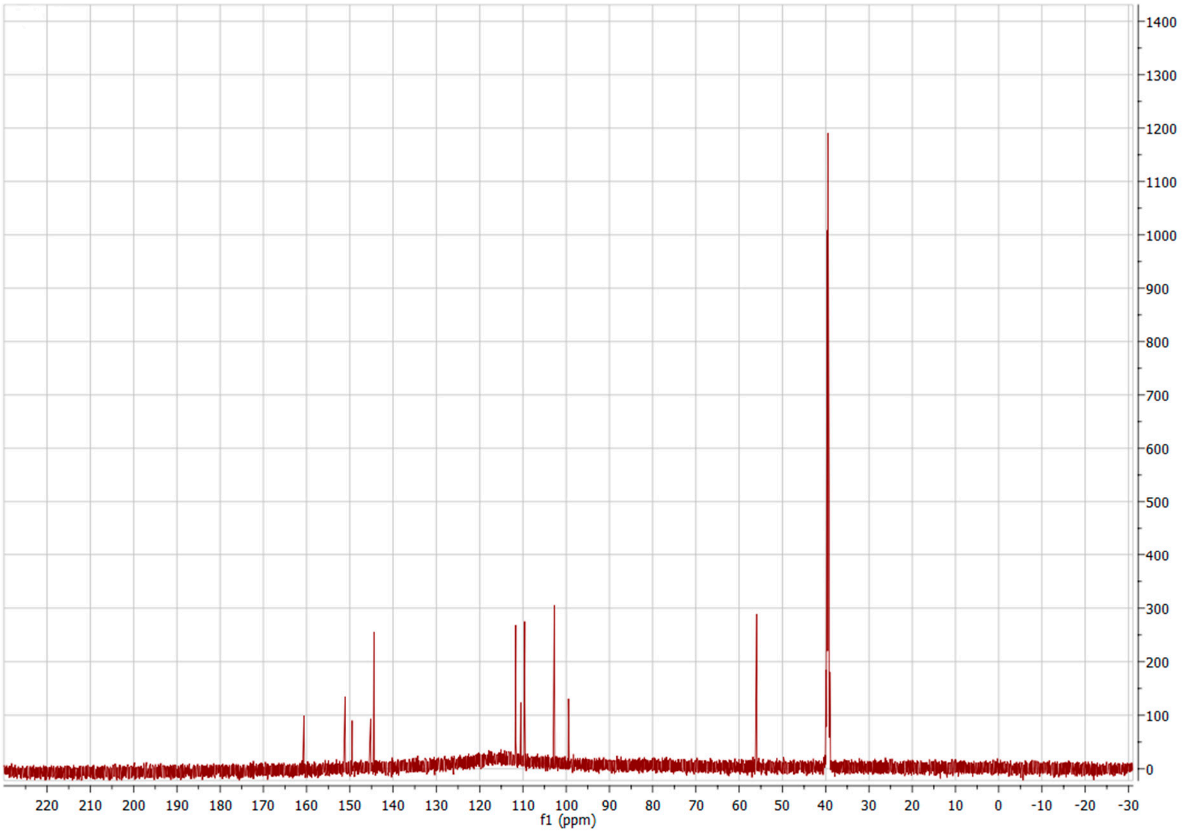

Pos.	**29** δ_H
2	-
3	6.22 d (9.4)
4	7.91 d (9.4)
5	7.22 s
6	-
7	-
8	6.78 s
9	-
10	-
6-OCH_3	3.82 s
7-OCH_3	-

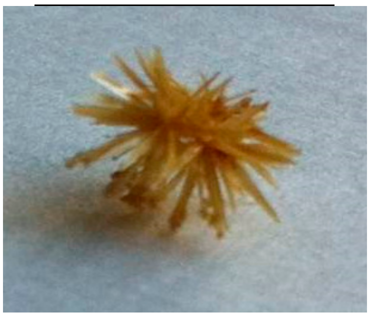

Crystals of scopoletin

Figure S3. X-ray analysis of β-arbutin (**42**) from *S. quinquefolia* leaf.

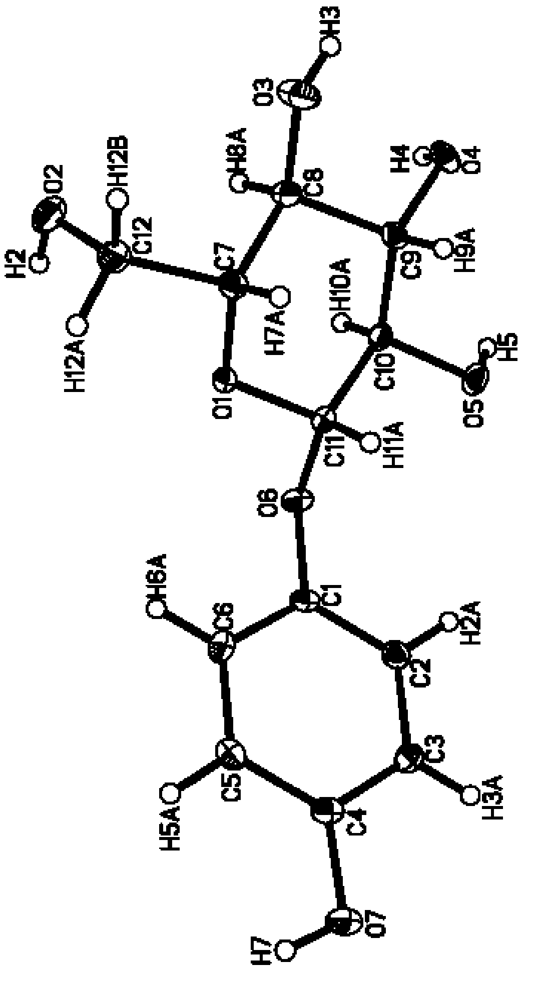

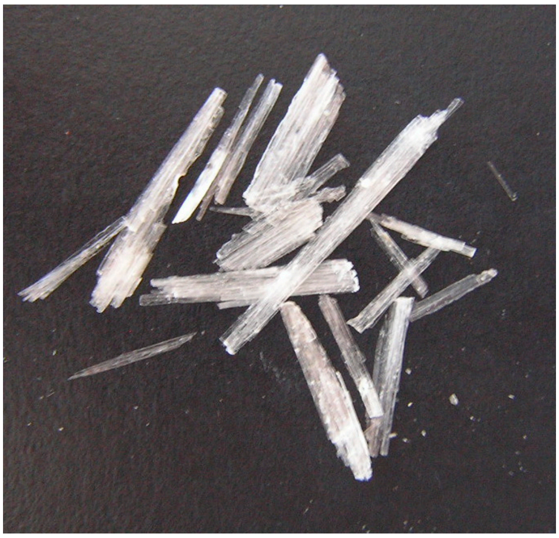

Figure S4. *Candida glabrata, Trichophyton rubrum, Microsporum canis, Scopulariopsis brevicaulis* cultures

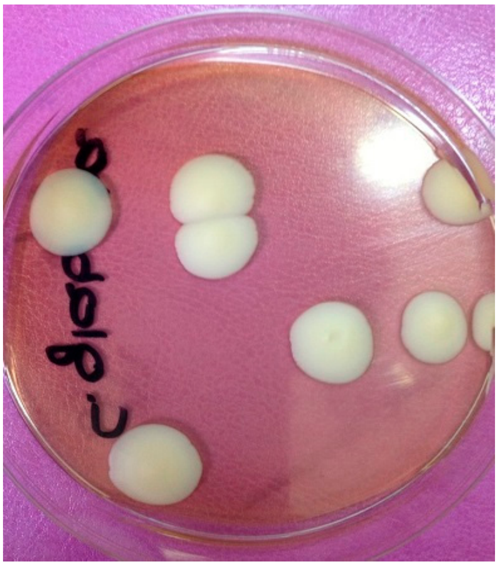

Candida glabrata

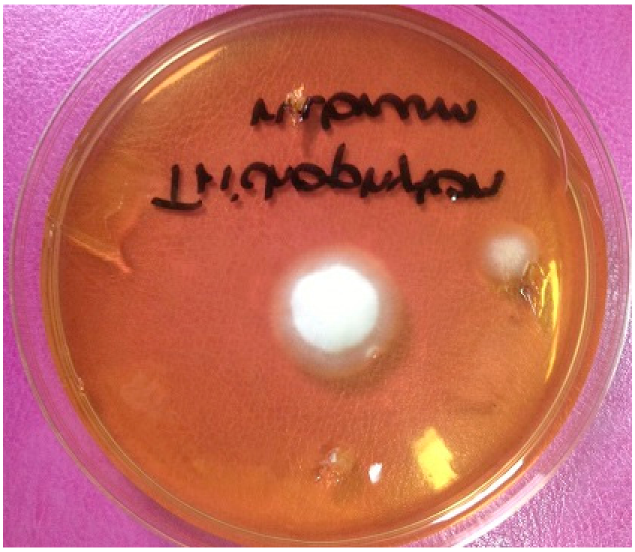

Trichophyton rubrum

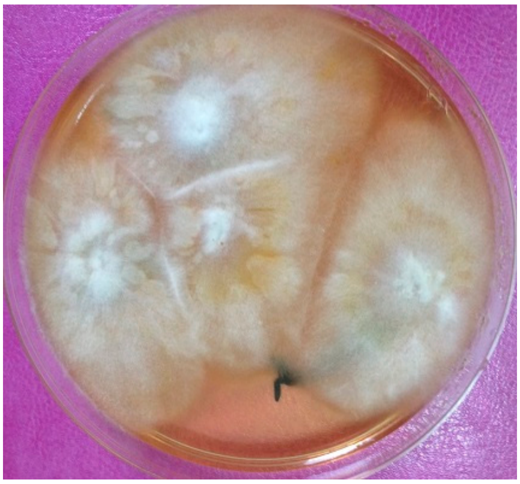

Microsporum canis

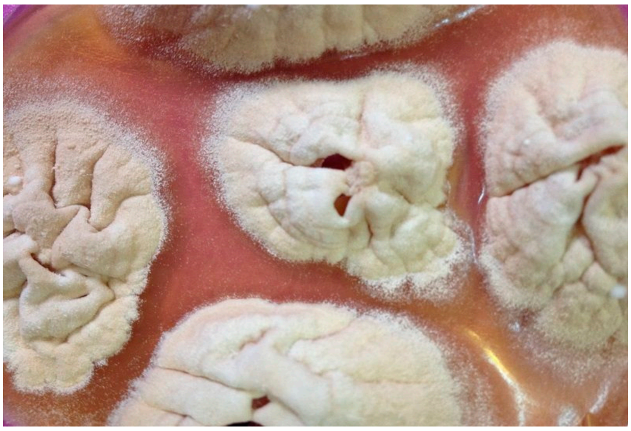

Scopulariopsis brevicaulis

Figure S5. TLC of lipophilic compounds from *Psephellus bellus* herb; Mobile phase: hexane – CH_2Cl_2 – AcOEt 4:2:5.

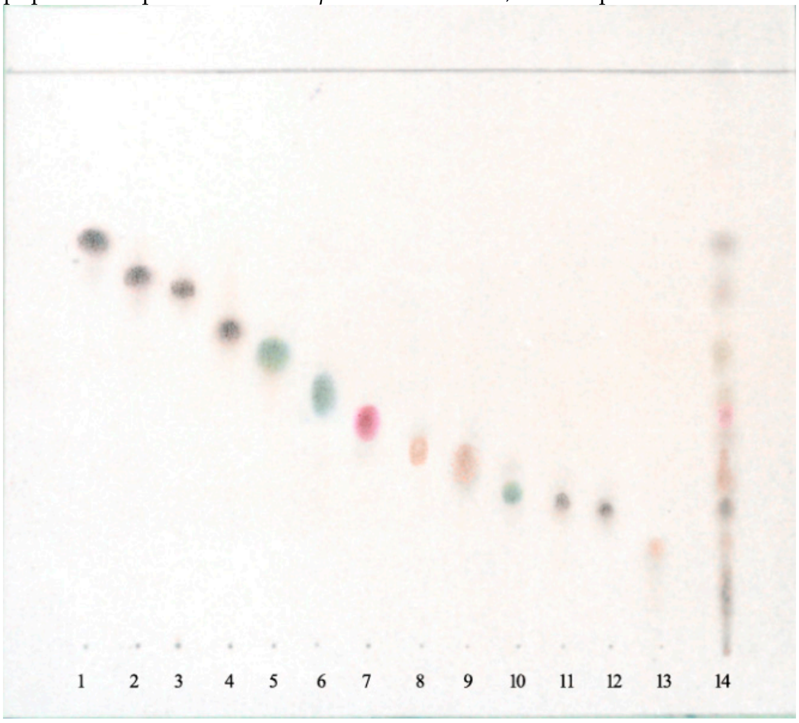

1. cebellin L; 2. cebellin O; 3. cebellin K; 4. cebellin N; 5.19-deoxychlorojanerin; 6. 17,18-epoxy-19-deoxychlorojanerin; 7. cebellin M; 8. 8-desacylo- -2′-methyl-acryloxy) subluteolide; 9. repin; 10. centaurepensin; 11. cebellin A; 12. cebellin B; 13. acroptilin; 14. extract from *Psephellus bellus* herb.

TLC method was used to assess the chemical composition of the plant extracts to plan a strategy of the separation of the compounds using the method of column chromatography (CC) (Figures 1, 2 Figures S2-S3). Moreover bases on TLC structures of sesquiterpene lactones could be rationalized with high probability. Black spots indicate guaianolides with the ester at C2 (compounds **1-4, 11**, **12**); the green colour suggest guaianolides with the chloromehyl group at C4 and hydroxyl at C3 (compounds **5** ,**6**, **10**); the brown colour is related to the presence of guaianolides with 4,5 epoxide and OH at C3 (compounds **8**, **9**, **13**); **7** as the only germacranolide has purple colour similar to some spots of germacranolides, with the substituent at C8, from *St. balsamita.*

Figure S6. TLC of coumarins from *Psephellus sibiricus* leaf

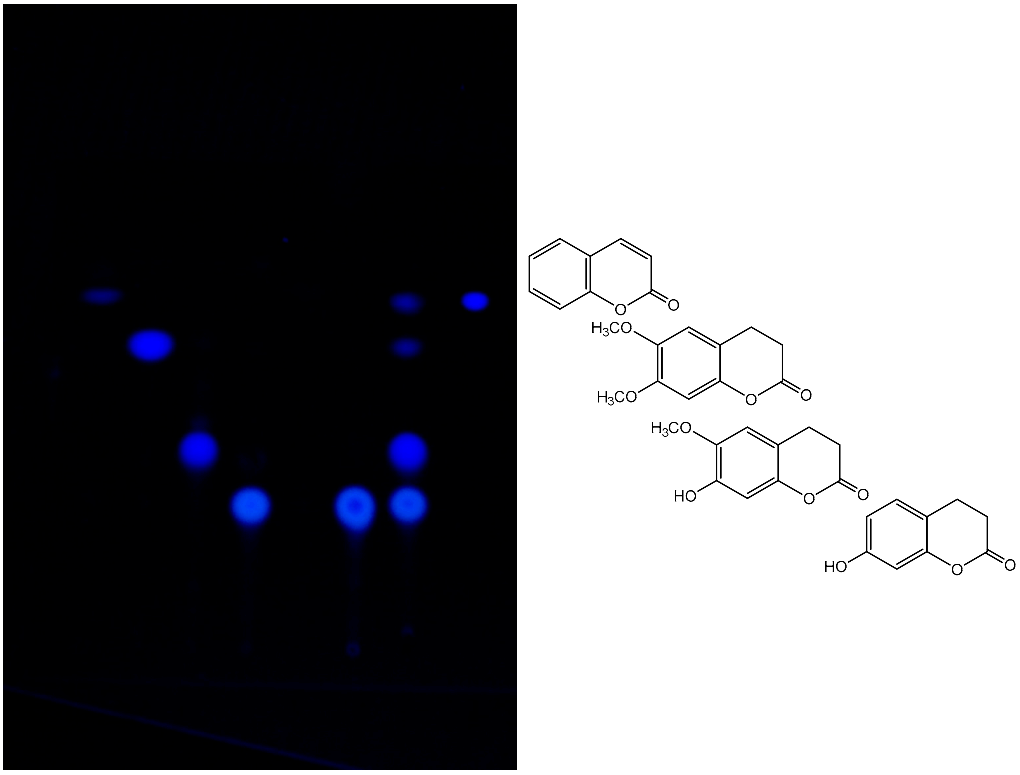

1 2 3 4 5 6 7

Mobile phase: CH_2Cl_2 – $CH_3(CO)_2$ 15:1 1. coumarin, 2. scoparone, 3. scopoletin, 4. umbelliferone, 5. umbelliferone (sample), 6. The extract from *P. sibiricus* leaf 7. coumarin (sample).

Figure S7. The HPLC chromatogram of water extract from *S. quinquefolia* leaf.

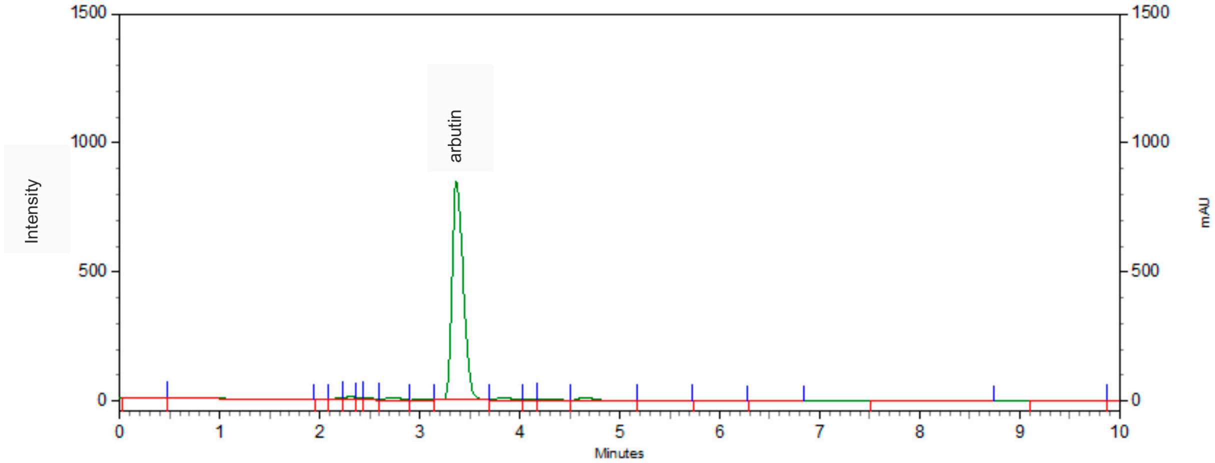

Preparation of the test sample solution: 0.4 g of dried *Serratula quinquefolia* leaf was weighed into a flask of 50 mL capacity, and 30 mL of 10% MeOH was added and placed in an ultrasonic bath for 5 min. Mixed and filled with a solvent, the resulting solution was filtered through a 0.45-lm membrane filter (Schleider & Scheuller).
